# Insoluble solids at high concentrations repress yeast’s response against stress and increase intracellular ROS levels

**DOI:** 10.1038/s41598-019-48733-w

**Published:** 2019-08-22

**Authors:** Antonio D. Moreno, Cristina González-Fernández, Mercedes Ballesteros, Elia Tomás-Pejó

**Affiliations:** 1 CIEMAT, Department of Energy, Biofuels Unit, 28040 Madrid, Spain; 2 IMDEA Energy Institute, Biotechnological Processes Unit, 28935 Móstoles, Spain

**Keywords:** Industrial microbiology, Applied microbiology

## Abstract

Lignocellulosic ethanol production requires high substrate concentrations for its cost-competitiveness. This implies the presence of high concentrations of insoluble solids (IS) at the initial stages of the process, which may limit the fermentation performance of the corresponding microorganism. The presence of 40–60% IS (w/w) resulted in lower glucose consumption rates and reduced ethanol volumetric productivities of *Saccharomyces cerevisiae* F12. Yeast cells exposed to IS exhibited a wrinkled cell surface and a reduced mean cell size due to cavity formation. In addition, the intracellular levels of reactive oxygen species (ROS) increased up to 40%. These ROS levels increased up to 70% when both lignocellulose-derived inhibitors and IS were simultaneously present. The general stress response mechanisms (e.g. *DDR2*, *TPS1* or *ZWF1* genes, trehalose and glycogen biosynthesis, and DNA repair mechanisms) were found repressed, and ROS formation could not be counteracted by the induction of the genes involved in repairing the oxidative damage such as glutathione, thioredoxin and methionine scavenging systems (e.g. *CTA1*, *GRX4*, *MXR1*, and *TSA1*; and the repression of cell cycle progression, *CLN3*). Overall, these results clearly show the role of IS as an important microbial stress factor that affect yeast cells at physical, physiological, and molecular levels.

## Introduction

Bioethanol fuel has increased in many countries as an effective alternative to reduce CO_2_ emissions in the transport sector. The traditional technology for converting sugar- and starch-based feedstocks into ethanol is relatively simple. However, advanced biofuels, such as lignocellulosic bioethanol, hold more opportunities for unlocking the potential of biofuels in order to achieve climate mitigation targets. Although lignocellulosic ethanol started to be commercialized by Beta Renewables back in 2013^1^ and several companies have built different industrial-scale plants since then (mainly in the US)^2^, the technology is still being developed due to the high recalcitrant structure of lignocellulose. Such limitation hinders the cost-competitiveness of lignocellulosic ethanol, making necessary further optimization of the biomass processing and microbial conversion steps to implement a fully effective technology.

Simultaneous saccharification and fermentation (SSF) and consolidated bioprocessing (CBP) have appeared as very attractive options for a cost-effective lignocellulosic ethanol production^3,4^. These strategies benefit from the integration of saccharification and fermentation in a single stage, and the tremendous overall cost savings due to the *on-site* enzyme production^5^. Working at high substrate loadings is also crucial for a cost-effective ethanol production, since this strategy minimizes distillation costs and reduces freshwater requirements. However, the complexity of the lignocellulosic fermentative medium in the form of solid materials and inhibitory biomass-derived compounds (phenolic compounds, furan derivatives and low molecular weight aliphatic acids) increases at high substrate concentrations, making microbial robustness of utmost importance^6^. The effects of biomass degradation compounds on microbial fermentation performance have been widely studied to understand their inhibitory mechanisms and overcome them^7–9^. However, the potential effects of insoluble solids (IS) (i.e., the water insoluble solids (WIS) fraction from pretreated lignocellulosic biomass) on fermentative microorganisms have been constantly undervalued.

The presence of high concentrations of IS at early stages of SSF and CBP processes may represent an important stress factor for fermentative microorganisms, affecting the fermentation performance and/or the tolerance to biomass-derived inhibitors^10,11^. Friction and/or collision mechanisms between IS and cells occur during mixing and, in consequence, cell morphology may be deformed, even causing the disruption of cell membranes and/or cell death. On the other hand, the effect exerted by the IS might indirectly modulate the gene expression pattern, inducing metabolic changes and altering microbial performance in terms of sugar conversion and/or inhibitory tolerance. Elucidating the variations in gene expression and determining the physiological consequences of IS on fermentative microorganisms are therefore necessary to direct future research actions for the development of more robust strains for the ethanol industry.

The present work evaluates the effects exerted by IS on *Saccharomyces cerevisiae* F12, focusing in aspects such as fermentation performance, cell viability, cell morphology, accumulation of reactive oxygen species (ROS), and the differences on the gene expression pattern. These results will boost the setting of the physiological and molecular basis towards a comprehensive understanding of the mechanisms beyond the effects exerted by IS on yeast cells, which has been often underestimated.

## Materials and Methods

### Microorganism and preinoculum growth

*S. cerevisiae* F12 was used as fermentative microorganism^12^. This strain presents an industrial background and it was genetically modified for lignocellulosic bioethanol production. Besides, it has been successfully used in bioethanol production processes from lignocellulose^13^. Active cell cultures were obtained by growing one single colony in 100-mL shake flasks with 20 mL YPD medium (10 g/L yeast extract, 20 g/L peptone, 20 g/L glucose). Cells were incubated in an orbital shaker at 32 °C and 150 rpm for 18 h. Then, cells were harvested by centrifugation (3000 g, 8 min, 25 °C) and diluted with the corresponding medium to get the appropriate inoculum size.

### Fermentation tests

The influence of IS on the yeast fermentation capacity was evaluated by subjecting *S. cerevisiae* F12 to fermentation in the presence of increased concentrations of solids. Fermentation assays with 0%, 40% and 60% IS (w/w) were performed in 250-mL shake flasks with 100 mL YNB media (Conda, Cat.1553.00) supplemented with 20 g/L glucose and 7.5 g/L (NH_4_)_2_SO_4_. After inoculation (0.5 g/L dry weight (DW)), cells were incubated in an orbital shaker at 32 °C and 150 rpm for 48 h. Samples were periodically withdrawn for determination of extracellular metabolites and cell viability.

Degradation compounds embedded in lignocellulosic WIS fractions may interfere with the RNA extraction procedure^14^, which might lead to misleading conclusions. To ease yeast separation and avoid interferences with analytical methods, 4-mm diameter glass beads (Hecht Karl™ 1401/4) were used as IS source instead of pretreated lignocellulosic fibers. The particle size of glass beads was within the common range of pretreated lignocellulosic biomass^15^.

Glucose and ethanol were analyzed by high performance liquid chromatography (HPLC) (Agilent infinity 1260, equipped with a refractive index detector), using an Aminex HPX-87H Ion Exclusion column (50 °C) with 5 mM H_2_SO_4_ (0.6 mL/min) as mobile phase.

Cell viability was measured with the Vi-Cell^TM^ XR analyzer (Beckmann Coulter).

Statistical analyses were performed using IBM SPSS Statistics v22.0 for MacOs X Software (SPSS Inc.). The mean and standard deviation were calculated from triplicates for descriptive statistics. When appropriate, analysis of variance (ANOVA) with or without Bonferroni’s post-test was used for comparisons between assays. The level of significance was set at 95%, 99% or 99.9%.

Intracellular glycogen and trehalose were measured on cells collected after 6 h and 24 h of fermentation in the presence of 0% and 40% IS (w/w), following the protocol described by Nielsen *et al*.^16^.

### Atomic force microscopy

Atomic force microscopy (AFM) was used to visualize the effects promoted from direct cells-solids interaction. Cells collected after 4 h of fermentation were first immobilized by mechanical trapping into 25-mm porous polycarbonate membranes (Nuclepore 1 µm, Whatman), and air-dried for 20 min prior to attaching it in the specific AFM liquid cell. AFM measurements were performed at room temperature in a Park XE-100 AFM (Park Systems), using dynamic contact mode and constant height screening. The maximum range for the XY scanner was 100 µm to allow the screening of 90 × 90 µm^2^. The maximum shift of *z* was −6 µm +6 µm. Differences in topography higher than 12 µm were not determined. Morphological changes on the yeast cell surface due to the presence of IS were visualized by using non-contact Si_3_N_4_ cantilevers (NSG30, NT-MDT) with a nominal spring constant of 40 N/m, high resonance frequency (320 kHz) and curvature radius lower than 10 nm.

### ROS measurement

Intracellular ROS concentration was determined in cells collected (3000 g, 8 min, 25 °C) after 4 h of fermentation under the following conditions: (1) absence of IS, (2) presence of 40% IS (w/w), (3) presence of 12.5% (v/v) lignocellulose-derived inhibitors, and (4) presence of 12.5% (v/v) lignocellulose-derived inhibitors and 40% IS (w/w). The inhibitor mixture (1.5 g/L furfural, 0.4 g/L 5-HMF, 6 g/L acetic acid, 2.5 g/L formic acid, 0.06 g/L ferulic acid, 0.03 g/L syringaldehyde, 0.1 g/L vanillin, and 0.05 g/L coumaric acid) was prepared according to compounds and concentrations commonly found in steam-exploded lignocellulosic hydrolysates^13^.

ROS accumulation was monitored by flow cytometry using dihydroethydium (DHE) as the ROS indicator. Cell pellets of about 10^6^ cells were obtained and diluted with 0.5 mL PBS buffer. Then, DHE was added to a final concentration of 2 µM, and the mixture was incubated for 30 min at 37 °C in dark conditions. DHE permeated into cells and gets oxidized to ethidium when exposed to superoxide in a dose-dependent manner. Ethidium then intercalates with DNA and emits red fluorescence proportional to intracellular ROS^17,18^. A blue laser (488 nm) was used for the excitation, and DHE emission was collected at 585/40 nm. A negative control with non-dyed cells was used as process checkup. Analysis was performed with a Cytomics FC 500 cytometer (Beckman Coulter) equipped with an FL3 detector (620 BP). In total, 50,000 cells were collected. The specific intracellular ROS level was obtained by normalizing the intracellular ROS concentrations with a negative control of cells non-treated with DHE.

### Microarray analysis of differential gene expression

Total RNA was extracted from cells after 4 h of fermentation in the presence and absence of 40% IS (w/w). 5-mL samples were withdrawn, cooled on ice, centrifuged (4000 g, 2 min, 4 °C), and cell pellets were rapidly frozen in liquid nitrogen and stored at −80 °C until analysis. Total RNA was isolated using Trizol reagent (Invitrogen) according to the manufacturer’s protocol, and treated with RNase-free DNase I (Qiagen) to prevent DNA contamination. The concentration and purity of RNA was measured using Omega spectrophotometer. RNA integrity was determined using Bioanalyzer 2100 (Agilent). Only those samples with 260/280 > 1.8; 260/230 > 2.0; and RNA Integrity Number (RIN) > 8.0 were further analyzed.

Equal amounts of each RNA sample were retro-transcribed to cDNA using random sequence oligonucleotide hexamers as primers. Template RNAs were then degraded with NaOH and cDNAs were labeled using TdT DNA polymerase and ddUTP-biotin. Labeled cDNAs were processed with GeneChip® IVT PLUS Reagent Kit (Affymetrix®), hybridized with GeneChip™ Yeast Genome 2.0 Array (Affymetrix®) and scanned with a GeneChip® Scanner 3000 7 G (Affymetrix®). Raw data were processed with RMA algorithm included in Affymetrix® Expression Console™ for normalization and gene level analysis. For each experimental condition, three microarray experiments corresponding to three independent RNA replicates were processed and analyzed. First, fold changes between experimental conditions were calculated as a quotient between the mean of the gene expression signals. Statistical analysis was performed with LIMMA package included in Babelomics software package [http://www.babelomics.org]^19^. Those values with a false discovery rates (FDR) < 0.05 were considered as significant. Genes with Log2-fold change >1 or <(−1) were included for further analysis. Furthermore, microarray experiments were analyzed by Piano software [http://biomet-toolbox.chalmers.se]^20^. In this case, FDR < 0.001 was used as gene selection cut-off in order to identify those differentially expressed genes with a higher statistical significance. Values corresponding to FDR < 0.05 were used for obtaining the corresponding heat map.

Microarray data were submitted to the NCBI GEO with GSE115460 as accession number [https://www.ncbi.nlm.nih.gov/geo/query/acc.cgi?acc=GSE115460].

Differentially expressed genes were classified by YeastMine according to their main known/proposed functions^21^. Thus, downregulated and upregulated genes were used to investigate and categorize the gene ontology (GO)-annotations, including both biological processes and molecular functions. Finally, network analysis of known/predicted protein-protein interactions was evaluated using STRING software v10.5^22^.

## Results

### Insoluble solids reduce cell viability affecting the fermentation performance

**Figure 1:**
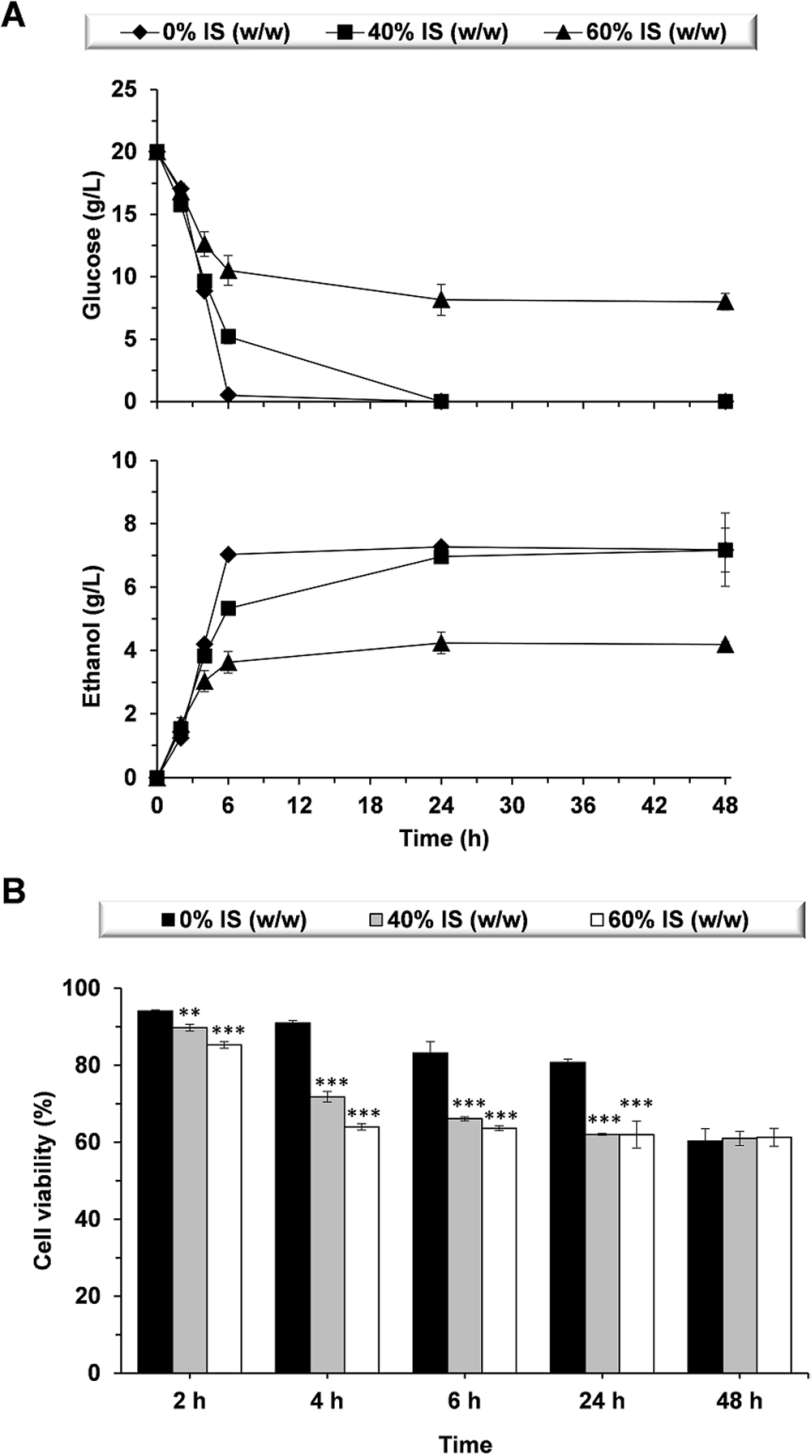
Fermentation performance of *S. cerevisiae* F12 in the presence and absence of insoluble solids (IS). Time-course for (**A**) glucose consumption and ethanol production, and (**B**) cell viability. Significant differences: **P < 0.01, ***P < 0.001.

Fermentation tests with 40% and 60% IS (w/w) were performed to envisage any effect exerted by IS on the ethanol production of *S. cerevisiae* F12. When using pretreated lignocellulosic biomass, substrate concentrations up to 40% (w/w) dry matter (DM) have been previously reported^23^. When compared to glass beads, the water retention capacity of biomass reduces the water available for yeast cells during the initial stages of the fermentation processes. In this context, a higher content of glass beads might simulate such effect and, therefore, a concentration of 60% IS (w/w) was also investigated. The presence of IS during the fermentation process resulted in lower glucose consumption rates and reduced ethanol volumetric productivities at early times, independently of the IS concentration (Fig. 1A). 5.2 ± 0.6 and 10.5 ± 1.2 g/L of glucose remained in the media after 6 h of fermentation in presence of 40% and 60% IS (w/w), respectively, while total glucose depletion was observed in absence of IS (w/w) (Fig. 1A, Table 1). The decrease in glucose consumption rates led to lower ethanol concentrations and lower ethanol volumetric productivities. Ethanol volumetric productivities were 1.2 ± 0.0, 0.9 ± 0.0 and 0.6 ± 0.1 g/L h for 0%, 40%, and 60% IS (w/w) assays, respectively (Fig. 1A, Table 1). In this context, Fig. 1B clearly shows a 20–30% reduction (*P* *<* 0.001) in cell viability due to the presence of 40% or 60% IS (w/w) at 6 h of process, thus explaining the lower glucose consumption rates and the reduced ethanol volumetric productivities when compared to 0% IS (w/w). However, the absence of differences in viability when comparing 40% and 60% IS (w/w) does not explain the disparity in glucose consumption at 6 h, which may be connected to metabolic and gene expression changes.

**Table 1:** Fermentation parameters in presence and absence of insoluble solids (IS).

	Ethanol_max_ (g/L)	Yield_E_ (g/g)^a^	Q_E_ (g/L h)^b^	Glucose_6h_ (g/L)^c^
0% IS (w/w)	7.3 ± 0.0	0.37 ± 0.00	1.2 ± 0.0	0.0 ± 0.0 (0%)
40% IS (w/w)	7.0 ± 0.1	0.35 ± 0.00	0.9 ± 0.0	5.2 ± 0.6 (26%)
60% IS (w/w)	4.3 ± 0.3	0.22 ± 0.01	0.6 ± 0.1	10.5 ± 1.2 (53%)

^a^Ethanol yields were determined as follows: [Ethanol_max_]/[glucose_initial_].

^b^Ethanol volumentric productivities were estimated at 6 h time point as follows: [ethanol_6h_]/t.

^c^Percentages from the initial glucose concentration are indicated in brackets.

The final ethanol concentration was clearly dependent on the IS content (Fig. 1A, Table 1). In presence of 40% IS (w/w), similar ethanol concentrations (7.0 ± 0.1 g/L) to those observed in the absence of solids (7.3 ± 0.0 g/L) were attained at 24 h. However, due to the lower cell viability and the lower glucose consumption rates, the peak for maximum ethanol concentration was delayed when 40% IS (w/w) were present (Fig. 1A). In the presence of 60% IS, maximum ethanol concentration was 4.3 ± 0.3 g/L (w/w) (Fig. 1A, Table 1), which represents a 42% reduction (*P* *<* 0.001) when compared to 0% and 40% IS (w/w). In all cases, the ethanol concentration remained constant up to 48 h of process (Fig. 1A), and there was no glucose consumption after 24 h in presence of 60% IS (w/w), even though cell viability was maintained above 60% (Fig. 1B) with a total cell population of 42.4 ± 2.6 Mcells/mL (52.9 ± 1.4 Mcells/mL were observed at 24 h).

### Insoluble solids directly promote changes in cell morphology and increase intracellular ROS

**Figure 2:**
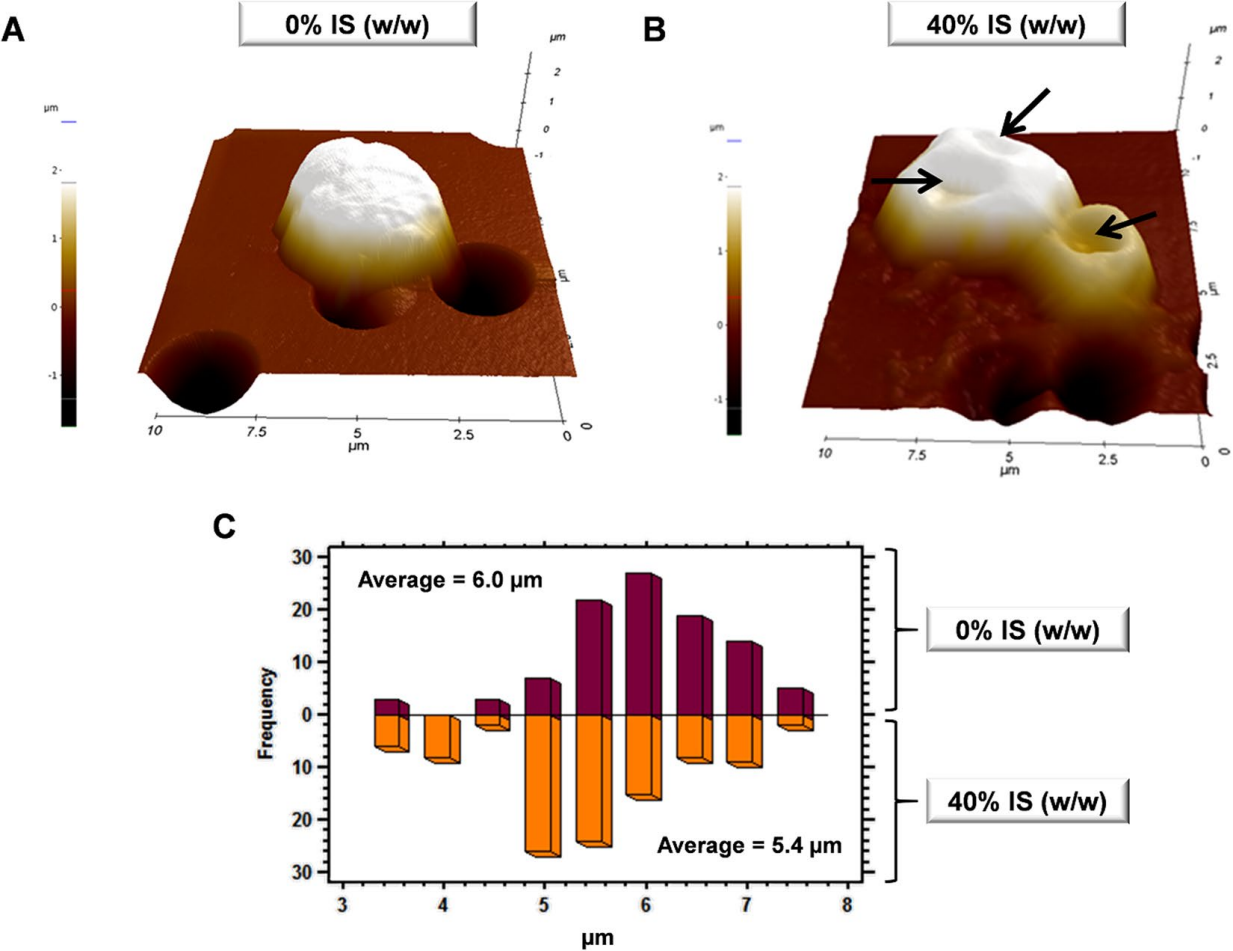
AFM imaging of *S. cerevisiae* F12 exposed to 0% and 40% (w/w) insoluble solids (IS). (**A**,**B**) 3D-reconstruction from 10 μm x 10 μm AFM topographic images. (**C**) Cell size distribution in presence and absence of IS. Arrows are indicative of cavity formation.

Cells exposed to an external chemical or physical stressor have previously shown important changes on cell surface^24,25^. To determine any variation on the cell surface and size, cells collected after 6 h of fermentation in the presence and absence of IS were visualized by AFM, since the yeast performance was severely affected at this time point. As shown in Fig. 2, cells markedly changed their cell surface topography from a round-turgid shape in the absence of IS (Fig. 2A) to a highly wrinkled morphology when solids were present (Fig. 2B). These changes were mainly promoted by the formation of cavities, which also caused a reduction in the mean cell size from 6.0 to 5.4 µm (*P* *<* 0.001) (Fig. 2C).

**Figure 3:**
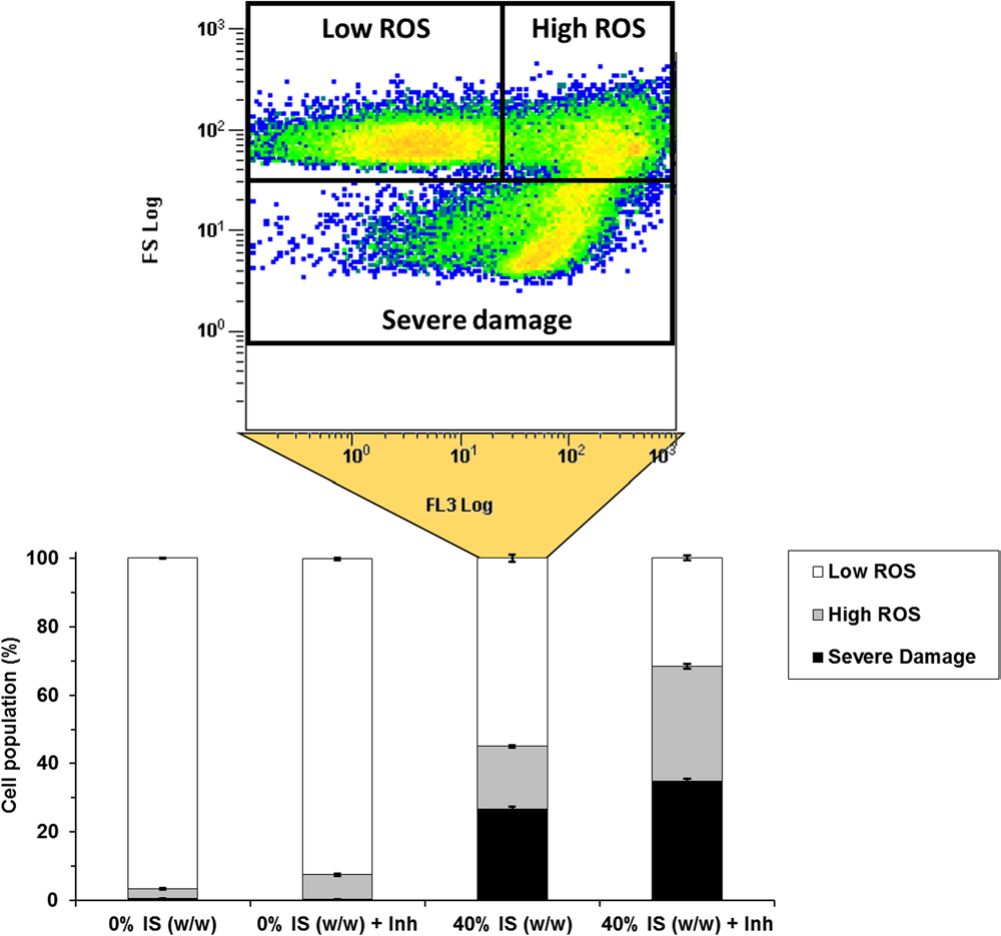
Accumulation of reactive oxygen species (ROS) in *S. cerevisiae* F12 in presence and absence of 40% (w/w) insoluble solids (IS), lignocellulosic-derived inhibitors (Inh), and both IS and Inh. Raw data from sample 40% IS (w/w) are visualized as an example for populations grouping. Low ROS cells were defined as cells adjusted to the negative control. High ROS cells were assigned to those reaching the highest levels of fluorescence. High levels of ROS together with low cell sizes were assigned to cells presenting severe damage.

ROS accumulation is another effect typically induced by exogenous stress factors^26^. In this work, ROS accumulation was evaluated in the presence of 40% IS (w/w) after 4 h of fermentation. The time point was chosen according to the better viability observed (ROS content is evaluated on viable cells) when compared to the 6-h time point (Fig. 1B). Low ROS levels were measured in the absence of IS. In contrast, above 45% (*P* *<* 0.001) of the viable cell population subjected to fermentation in the presence of 40% IS (w/w) showed high ROS levels, with almost 30% of them evidencing severe damage (*P* *<* 0.001) (Fig. 3). With the aim of evaluating any potential synergy with lignocellulosic-derived compounds, ROS levels were also measured in cells growing in the presence of inhibitors with or without IS. The cell population subjected to fermentation in presence of 12.5% (v/v) lignocellulose-derived compounds showed, in general, low ROS levels in the absence of IS. Nonetheless, the cell percentage showing high ROS levels increased up to 70% (P < 0.001) (35% of which showed severe damage) when IS and lignocellulose-derived inhibitors were simultaneously present.

### Gene expression pattern in the presence of insoluble solids

**Figure 4:**
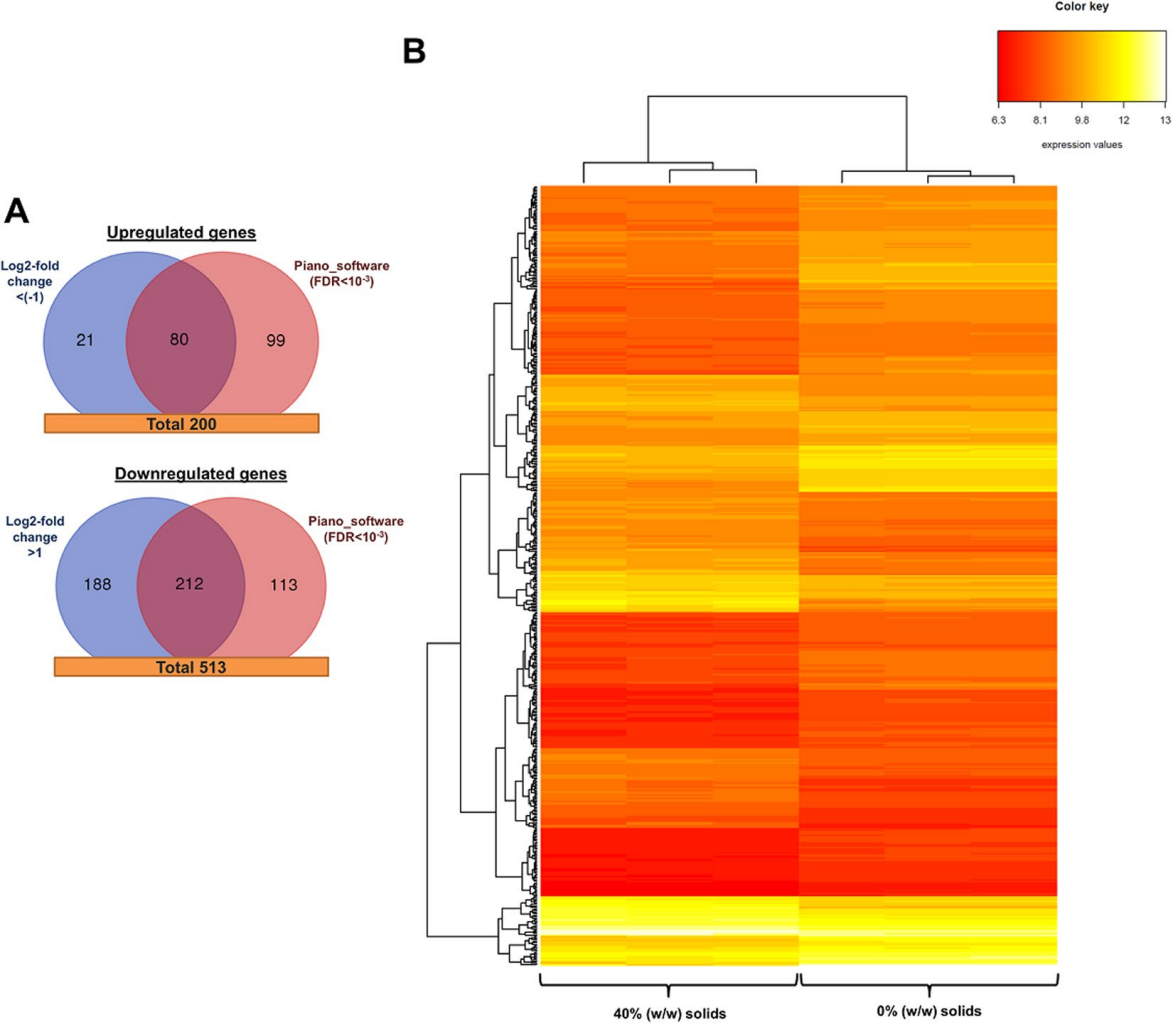
Differentially expressed genes in presence of 40% (w/w) insoluble solids (IS). (**A**) Venn diagrams of upregulated and downregulated gene numbers identified by the Log2-fold change and Piano software. (**B**) Heatmap and hierarchical clustering of the differentially expressed genes.

The presence of IS might also influence yeast cell physiology by modulating the gene expression pattern. Differential expression analysis (FDR < 0.05) identified 200 genes overexpressed and 513 genes downregulated after 4 h of fermentation in the presence of 40% IS (w/w) (Fig. 4A). These values represent the 11% equivalent of the total gene number in *S. cerevisiae* (6433 genes) [https://www.yeastgenome.org]. Differences in gene expression were identified by using the Log2-fold change method^27^ and Piano software^20^ (FDR < 0.001), showing above 40% matches (292 genes) between them. In addition, a heatmap using hierarchical clustering was built based on differentially expressed genes (FDR < 0.05) with the aim of grouping samples with similar data. This plot clearly identified two different clusters (Fig. 4B): (i) one corresponding to cells collected during fermentation in presence of 40% IS (w/w), and (ii) one corresponding to cells collected in absence of solids. These results support the differences on the gene expression pattern between cell populations.

The main biological processes influenced by the presence of IS were determined by gene ontology analysis. Table 2 shows the enriched upregulated and downregulated biological processes, respectively (FDR < 0.05). Small molecule and sulfur metabolic processes, including amino acids biosynthesis, carboxylic acid metabolic processes, glutathione biosynthesis, etc., and processes involved in transportation and respiration were identified as major overexpressed biological processes in the presence of IS. In contrast, carbohydrate metabolic processes (trehalose and glycogen biosynthesis), cell response to stimulus (DNA repair, response to heat, desiccation or drugs) and certain general biological regulation mechanisms (regulation of gene expression, RNA biosynthesis, signal transduction, etc.) were, on the other hand, repressed by the presence of IS. Enrichment analysis of differentially expressed genes could also identify specific repressed and overexpressed (*P* *<* 0.01) metabolic pathways (Table 3). Thus, glycogen and trehalose biosynthetic pathways, and the oxidative branch of the pentose phosphate pathway were found to be downregulated, while sulfate assimilation pathway and superpathway of sulfur amino acid biosynthesis were upregulated in the presence of 40% IS (w/w). Downregulation of glycogen and trehalose biosynthetic pathways was also supported by actual metabolite concentrations (Supplementary Fig. S1). After 6 h of fermentation, trehalose content was below the detection limits, while glycogen content was 260 ± 18 and 147 ± 25 mg/g DM (*P* < 0.01) for 0% and 40% IS (w/w), respectively. After 24 h, trehalose content was 4.7 ± 1.3 and 0.5 ± 0.2 (*P* < 0.01) for 0% and 40% IS (w/w), respectively, while no differences on glycogen content were found (130–140 mg/g DM).

**Table 2:** Upregulated and downregulated biological processes enriched in the presence of insoluble solids (IS)

Biological Process	GO Term	Gene N°	*P*-value^a^
**Upregulated processes** **:**			
Small molecule metabolic process^1^	GO:0044281	58	6.10E-06
Sulfur compound metabolic process^2^	GO:0006790	21	1.53E-05
Oxidation-reduction process^3^	GO:0055114	38	1.88E-04
Drug metabolic process^4^	GO:0017144	27	3.35E-04
Transmembrane transport^5^	GO:0055085	37	6.31E-04
Cellular respiration^6^	GO:0045333	15	1.42E-02
**Downregulated processes** **:**			
Carbohydrate metabolic process^7^	GO:0005975	53	4.91E-05
Response to stimulus^8^	GO:0050896	148	6.84E-03
Pyridine-containing compound metabolic process^9^	GO:0072524	23	7.23E-03
Biological regulation^10^	GO:0065007	218	1.99E-02

^a^Multiple testing was analyzed by Holm-Bonferroni test.

^1^Amino acid metabolic process, organic acid metabolic process, sulfur compound metabolic process, TCA metabolic process, respiratory electron transfer chain.

^2^Sulfur compound metabolic process, methionine and cysteine biosynthetic process, drug metabolic process.

^3^Cellular respiration, TCA metabolic process, ATP synthesis coupled electron transport, sulfur metabolic process.

^4^Antibiotic metabolic process, allantoin metabolic process, toxin metabolic process, aerobic respiration, sulfate assimilation.

^5^Transport involving ions, organic acids, amino acids, and sulfate.

^6^Aerobic respiration, TCA metabolic process, electron transport chain.

^7^Hexose metabolic process, trehalose metabolic process, glycogen metabolic process, gluconeogenesis, pentose-phosphate pathway: oxidative branch.

^8^Cellular response to stress: chemical, drug, desiccation, temperature, oxidative stress, DNA repair, cell cycle.

^9^Coenzyme metabolic process, phosphorus metabolic process, vitamin metabolic process, ATP generation from ADP.

^10^Cell cycle, regulation of gene expression, ion homeostasis, signal transduction, regulation of primary metabolic process.

**Table 3:** Gene enrichment analysis of specific metabolic pathways repressed and overexpressed in the presence of insoluble solids

Metabolic Pathway	Pathway Ref.	Related Genes	P-Value^a^
**Repressed** **:**			
Glycogen biosynthesis	PWY3O-4031	GLC3, GLG1, GSY1, GSY2, PGM2, UGP1	3.35E-03
Trehalose biosynthesis	TRESYN-PWY	TPS1, TPS2, TPS3, TSL1	8.15E-03
Oxidative branch of the pentose phosphate pathway	OXIDATIVEPENT-PWY	SOL4, GND2, ZWF1	1.33E-02
**Overexpressed** **:**			
Sulfate assimilation pathway	PWY-781	MET3, MET5, MET10, MET14, MET16	1.08E-03
Superpathway of sulfur amino acid biosynthesis	PWY-821	HOM3, MET2, MET3, MET5, MET10, MET14, MET16, MET17, STR3	2.23E-03

^a^Multiple testing was analyzed by Holm-Bonferroni test.

It is important to highlight that most of the differentially expressed genes (194 out of 513 downregulated genes, and 53 out of 200 upregulated genes) had, however, an unknown molecular function, and 30–40% of them encoded a putative protein with unknown function (Supplementary Table S1). Above 80% and 60% of these repressed and overexpressed genes had a Log2-fold change above one order, respectively, which may indicate a potential role in the cell response to IS. Indeed, although having an unknown molecular function, the upregulated genes *CIR2*, *KNH1*, *MIX17*, *YDL012C*, and *YGR266W*, and the downregulated genes *BIR1*, *DDR2*, *EIS1*, *FUN19*, *IZH4*, *MTL1*, *RGI1*, *SPI1*, *STF2*, *TMC1*, *YBL111C*, *YDR391C*, *YIL108W*, *YJL144W*, *YKR011C*, *YLR149C*, and *YRO2* have been previously identified with a key role in the cell response to stress (Supplementary Table S1).

**Figure 5:**
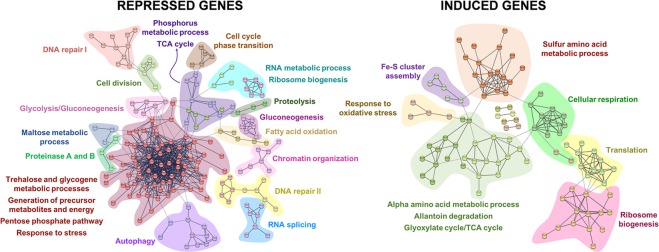
STRING analysis of differentially expressed genes in presence of 40% (w/w) insoluble solids (IS). Groups are based on the biological processes in which proteins are involved.

To bring more light in the differences found, differentially expressed genes were also analyzed by STRING software [http://string.embl.de/], which establishes the interaction between proteins that contribute together to the same function. Figure 5 illustrates the most significant protein-protein interactions (highest confidence score), grouped according to biological processes. Also, Table 4 shows the complete gene list resulted from STRING analysis. Overall, STRING and ontology analyses led to similar results, emphasizing the importance of protein metabolic processes, stress-response mechanisms, cell cycle regulation, and carbohydrate and lipid metabolic processes as the main biological processes influenced by the presence of IS.

**Table 4:** Grouped genes based on STRING protein-protein interaction analysis.

Biological Process	Genes^a^
**Upregulated genes** **:**	
*Alpha amino acid metabolic process, Allantoin degradation, Glyoxylate cycle/TCA cycle*	ACO1, ACO2, ARG1, ARG3, ARG5,6, ASN1, CAR1, CPA1, DAL1, DAL3, DAL4, DAL5, DAL7, ERV1, GLT1, HOM3, ICL1, IDH1, KAP123, LEU1, LEU4, LEU9, LYS1, LYS20, MIX17, PGA3
*Cellular respiration*	ACO1, COR1, COX7, CYC1, CYT1, ICL1, IDH1, MIX17, QCR2, SDH2, SDH3, SDH4
*Fe-S cluster assembly*	CFD1, CIA2, ISA1, NAR1, YAH1
*Response to oxidative stress*	CTA1, GPX2, GRX4, TSA1
*Ribosome biogenesis*	ALB1, BCP1, DHR2, DIM1, ECM1, EMG1, NHP2, NIP7, RLI1, RPL26B, RPS28A, RSM7, YTM1
*Sulfur amino acid metabolic process*	MET1, MET10, MET14, MET16, MET17, MET2, MET28, MET3, MET32, MET5, MET8, MHT1, MMP1, MXR1, STR3, SUL1, SUL2
*Translation*	CDC33, MNP1, MRPL27, MRPL28, MRPL32, NHP2, RLI1, RML2, RPL23B, RPL26B, RPL29, RPL9A, RPS28A, RSM7
**Downregulated genes** **:**	
*Autophagy*	APE1, ATG15, ATG17, ATG19, ATG20, ATG23, ATG29, ATG34, SNX4, SNX41, VPS41
*Cell cycle phase transition*	CLN3, MBP1, RLM1, SWI4, WHI5, (CDC7, CTR9, STE20, XBP1)
*Cell division*	CIN8, KRE28, NUF2, RNR3, SLK19, (ADY3, BIR1, BRN1, CDC37, CDC7, CLN3, CNM67, DON1, KAR1, MPC54, PDS5, SHS1, SMC2, SMC3, SMC4, SSD1, TPK1)
*Chromatin organization*	BRE1, CDC7, CTR9, ELF1, MCM10, POB3, RTF1
*DNA repair I*	BRN1, PDS5, RAD17, RAD50, RAD61, RAD9, SMC2, SMC3, SMC4, XRS2
*DNA repair II*	HFM1, MLH3, MSH2, MSH4, MSH5, RAD16, RAD2, RAD26, RAD7, UBP15
*Fatty acid oxidation*	FAA1, FAA2, POX1, PXA1, PXA2
*Gluconeogenesis*	FYV10, GID7, GPM2, MDH2, TDH1, UBC8, VID24, VID28, VID30
*Glycolysis/Gluconeogenesis*	PYK2, HXK1, TDH1, YOR283W, ERR3, GPM2
*Maltose metabolic process*	IMA1, MAL31, MAL32
*Phosphorus metabolic process, TCA cycle*	CIT3, CYR1, FMP48, GDH3, HFA1, IDP3, KDX1, MDH2, PBS2, PTK2, PTP2, SHH3, YBR139W, YJR149W
*Proteinase A and B*	PAI3, PEP4, PRB1
*Proteolysis*	PCI8, RPN1, RRI1, (FYV10, UBP15, UBP16, UBP2, UBP9, VID24, VID30, YGR066C)
*RNA metabolic process, ribosome biogenesis*	BUD22, KRI1, MAK16, NMD2, RRP14, RRP36, SAS10, TAP42, TOR2, UPF3, (REX3, RIO1, RPM1, SNU66, SQS1)
*RNA splicing*	CLF1, LIN1, PRP11, PRP3, SLU7, SNU66, SQS1, THP3, YJU2
*Trehalose and glycogene metabolic processes, generation of precursor metabolites and energy, response to stress*	ALD4, AQY2, ATH1, COX5B, DAK1, DCS1, DCS2, DDR2, ECM4, EGO4, EMI2, FMP33, FMP45, GAD1, GCY1, GDB1, GGA1, GIP2, GLC3, GLG1, GLK1, GND2, GOR1, GPD1, GPG1, GPH1, GSC2, GSY1, GSY2, HBT1, HSP104, HSP26, HSP42, HSP78, HXK1, HXT7, IGD1, MHO1, MSC1, NDE2, NTH1, OM45, PGM2, PNC1, RGI1, RTC3, RTN2, SBP1, SDS24, SFA1, SOL4, SPI1, SSE2, STF2, TFS1, TKL2, TMA17, TPK1, TPS1, TPS2, TPS3, TSL1, UGA1, UGP1, UIP4, YDL124W, YGP1, YJL144W, YLR149C, YLR345W, YPL247C, YRO2, ZWF1 (BIR1, EIS1, FUN19, GRX1, HSP30, HSP31, IZH4, MTL1, TMC1, TSA2, YBL111C, YDR391C, YIL108W, YKR011C)

^a^Other differentially expressed genes with similar function and not visualized by STRING analysis are indicated in brackets.

Protein metabolic processes modulated by the presence of IS included genes related to autophagy, protein degradation, and amino acid and protein metabolic processes. In this sense, differentially expressed genes related to autophagy (*ATG* genes) and protein modification/degradation mechanisms (including proteinases A and B) were both repressed by IS. Meanwhile, genes associated to amino acid biosynthesis (especially those encoding the synthesis of methionine and cysteine) and other protein metabolic processes, such as translation and ribosome biogenesis, were induced (Fig. 5, Table 4). It should be noted that *S. cerevisiae* F12 simultaneously repressed some of the genes needed for ribosome biogenesis. Besides, certain specific genes related to RNA processing, such as those encoding for the spliceosome, were also repressed, pointing out the high complexity of the cell response to the presence of IS.

Regulation of the cell cycle and the overall yeast stress response were also influenced by the presence IS. Thus, cells subjected to 40% IS (w/w) downregulated the expression of genes involved in cell division, cell cycle phase transition (including *CLN3*), chromatin organization, and different DNA recombination and repair mechanisms (Fig. 5, Table 4). Finally, about 30 genes involved in the yeast’s general response to stress were differentially expressed, including *DDR2*, *GPX2*, *GRX1*, *GRX4*, *HSP104*, *HSP30*, *HSP31*, *MTL1*, *TPS1*, *TSA1*, *TSA2*, and *ZWF1*, which are recognized as crucial genes to face heat, oxidative and/or osmotic stresses^28–31^. These genes were downregulated in most cases, including the specific genes related to trehalose and glycogen biosynthesis, two important carbohydrates involved in the cell response to stress^32,33^. In contrast, genes involved in the glutathione system, the assembly of Fe-S clusters, or the degradation of allantoin were induced.

## Discussion

The experience gained from brewing and starch-based fermentations under high gravity conditions have paved the way for the application of similar strategies during lignocellulose conversion processes. High gravity technologies are necessary to achieve high substrate concentrations, thus allowing high ethanol titers and reducing distillation costs^34^. At high substrate loading yeast cells have to deal with a high IS content during SSF/CBP processes, especially at early stages. Notwithstanding, when SSF/CBP proceeds liquefaction effect (due to enzymatic activity) that convert biomass particles into an aqueous paste takes part^23^. IS may produce stress and induce cell damage on fermentative yeasts^35^, although their potential consequences at early stages of SSF/CBP processes have been generally disregarded, and only the effects caused by biomass-derived inhibitors have been usually considered. In this work, *S. cerevisiae* F12 cells shrank and increased their surface roughness when exposed to IS, highlighting the role of IS as an important stressor during the fermentation processes. Differences in cell morphology have been previously observed when subjecting *S. cerevisiae* cells to heat^36^, osmotic^24^ and oxidative^25^ stresses, and even during the stress caused by the presence of high ethanol concentrations^37,38^. However, this is the first time that the changes in cell morphology are evidenced in the presence of IS. Osmostress, thermostress or oxidative stress have also shown to decrease mean cell volumes^24,25^. Similar observations were found in this work, where the mean cell size of cells exposed to IS was reduced by 10%. These results highlight the similarities between the effects promoted by solids and other physical and/or chemical stressors on cell surface.

In addition to induce morphological changes, IS have shown to influence the fermentation performance of yeast cells. The presence of 40% and 60% IS (w/w) decreased *S. cerevisiae* F12 viability by 20–30%, reducing the overall substrate consumption rates and ethanol volumetric productivities. Moreover, although cell population retained about 60% of cell viability in case of 40% and 60% IS (w/w) along the fermentation process, the highest IS content reduced final ethanol titers by 1.7-fold due to an incomplete glucose utilization. This result might be indicative of metabolic and gene expression changes occurring in cells subjected to 60% IS (w/w). Indeed, cells might be entering in a resting state when subjected to IS, since quiescence is typically induced to confer increased resistance to a wide range of environmental stress factors^39^. Supporting this hypothesis, 30 genes involved in the regulation of the cell cycle were found to be downregulated in the presence of IS at 4 h of process, including *CLN3*, which has been identified to be one of the earliest genes activated to promote the transition between G0 and G1 phases^40^. Quiescent or cell arrest is usually induced by nutrient starvation (e.g. glucose or nitrogen), and it is linked to the concomitant repression of ribosomal biogenesis genes and the induction of autophagy- and stress-responsive genes^41^. Ribosome synthesis is one of the major energy consuming processes of the cell, and it must be therefore limited under nutrient-limiting conditions^42^. Autophagy, on the other hand, is required to reallocating limited nitrogen through autophagic degradation of existing proteins and organelles, and/or for vacuolar nutrient sensing in mediating mitotic exit during nutrient starvation^43^. In this work, nutrient-rich conditions were used to evaluate the effects of IS on *S. cerevisiae* F12 cells, which might explain the induction of ribosome biogenesis and translation genes, and the repression of proteolysis and autophagy-related genes. Notwithstanding, ribosome synthesis is a highly complex process; therefore, certain genes involved in ribosome biogenesis were also found repressed in the presence of IS.

Cell arresting may also be promoted in response to stress-derived DNA damage in order to maintain genomic integrity of proliferating cells^44^. Optimal adaptation to stress involves an extensive reorganization of the gene expression^45^. In addition to repressed cell cycle transition, the presence of IS also influenced the expression of several genes related with the general cell response to stress. The expression of *DDR2*, *TPS1*, *HSP30*, *HSP104*, *MTL1*, *GRX1*, *TSA2*, and *ZWF1* is usually activated by a variety of chemical agents and environmental or physiological stresses^28,29,31,46^. For instance, the multi-stress response gene *DDR2* may be activated by more than 13 xenobiotic agents, heat shock, DNA damage and other stresses^28^. *TPS1* has been recently identified as a key gene for cell survival during heat stress, oxidative stress or desiccation^31^. The gene encoding for the negative regulator of the H(^+^)-ATPase (*HSP30*) and dissagregase protein (*HSP104*) are also overexpressed upon exposition to ethanol, heat, and osmotic stress^46^. On the other hand, *MTL1*, *GRX1*, *TSA2*, and *ZWF1* genes are usually overexpressed in the presence of chemical and oxidative stressors, including the lignocellulose-derived inhibitors furfural and 5-hydroxymethylfurfural^15,29,46,47^. The absence of proper sensing mechanisms for detecting the external damage promoted by IS might be behind the lack of an effective stress response, thus resulting in a defective activation of the corresponding protective mechanisms and increasing intracellular ROS levels. ROS are an unavoidable by-product of aerobic metabolism commonly accumulated as a consequence of cell exposure to different stressors^48^. In the presence of IS, about 40% of the cell population evidenced high ROS content. This population percentage was increased by 1.8-fold when combining IS and lignocellulose-derived inhibitors, even though these compounds caused minor ROS accumulation in the absence of IS. This fact highlights the serious consequences of having such environment during lignocellulose conversion processes at high substrate concentration.

Trehalose and glycogen biosynthetic pathways were also repressed in the presence of IS. These carbohydrates are two glucose storages of yeast cells and they accumulate in response to different environmental changes^32^. Furthermore, trehalose is one of the most effective substances known for preservation of membranous structures and enzyme activities during stress^33^. The lower trehalose levels combined with a high intracellular ROS content may synergistically contribute to increase cell damage, severely affecting DNA (promoting both base damage and strand breaks), proteins and other cellular components, and leading to cell membrane instability as well^26^. Therefore, the overall repression of these stress protective mechanisms besides the increase in ROS levels may negatively contribute to boost morphological changes on cell surface after the direct exposure to IS, also causing a drastic intracellular damage. Surprisingly, direct reversal repair pathway (involving *PHR1* gene), base excision repair pathway (involving *MAG1* gene), and RAD-related DNA repair pathways (nucleotide excision repair pathway, the homologous recombination pathway, and the postreplication repair pathway)^49^ were also downregulated in the presence of IS. Repression of these DNA repair pathways might be responsible for the decrease in cell viability, since all genome mutations caused by the high ROS levels would accumulate, ultimately promoting cell death^50^. This hypothesis is also supported by repression of *BIR1* (*BIR1* deletion mutants are more sensitive to apoptosis induced by oxidative stress) and *STF2* (deletion of *STF2* promotes the production of reactive oxygen species and apoptotic cell death during stress conditions) genes, since they have shown to be essentials for preventing the apoptotic mechanisms (programmed cell death) induced by oxidative stress^51,52^.

On the other hand, the presence of IS triggered the overexpression of the stress-related genes *CCP1*, *CIR2, CTA1*, *GPX2*, *GRX4*, *MXR1*, *POS5*, and *TSA1*. These genes encode different cytoplasmic and mitochondrial enzymatic activities, including cytochrome c peroxidase, dehydrogenase, catalase, glutathione peroxidase, oxidoreductase, glutaredoxin, kinase, and peroxiredoxin, as crucial antioxidant defenses^30,53,54^. These activities belong to the glutathione and thioredoxin systems that are responsible for maintaining redox homeostasis through complex regulatory machinery. Intracellular glutathione levels have shown to be essential for increasing *S. cerevisiae* robustness to the stress caused by lignocellulose-derived inhibitors^55^. In addition, previous biochemical studies have suggested that thioredoxin is the predominant antioxidant system in yeast, and also showing important interconnections with the glutathione system^30^. Both glutathione and thioredoxin systems are cysteine-demanding protective processes, since this amino acid residue is easily oxidized by ROS compounds. Cysteine, together with methionine, is a primary sulfur amino acid. Therefore, sulfate assimilation pathway and the superpathway of sulfur amino acid biosynthesis were induced in response to IS in order to support the synthesis of these compounds. The overexpression of these pathways may also involve a third antioxidant system, based on methionine amino acid. Similar to cysteine, methionine residues are extremely sensitive to ROS compounds, thus generating methionine sulfoxide. This oxidized methionine residue can subsequently be reduced through the catalysis of methionine sulfoxide reductases that are encoded by *MXR1* (which was also included within the overexpressed genes) and *MXR2*^56^, working as a natural scavenging mechanism to remove ROS compounds. Although these results seem to be contradictory with repression of *BIR1* and *STF2*, it is important to consider that RNA was likely collected from a population with cells at different metabolic stages due to the presence of an external stressor. This could be also inferred from the fact that viable cells remain almost constant at 6, 24 and 48 h when 40% or 60% IS (w/w) were present (RNA was collected from cells after 4 h of fermentation). Then, cell death might be more severe within 0–6 h, while remaining cells might enter in a quiescent state (as suggested by the downregulation of cell cycle) towards increasing its robustness under these conditions. Induction of stress-related mechanisms such as glutathione, thioredoxin and methionine-based scavenging systems might therefore have an important role in ROS protection and viability maintenance in presence of IS. Also, other non-identified mechanisms (e.g. changes in yeast cell wall) might also play a crucial role towards facing IS-induced stress.

In brief, the present study underlines the presence of high IS content as an important stress factor that promotes physical, physiological and genetic changes on fermentative microorganisms. Cells respond to IS with membrane wrinkling and deformation, as seen with other physical and/or chemical stressors (e.g. osmotic stress, desiccation, ethanol, etc.). Several multi-stress response genes are repressed, leading to the accumulation of intracellular ROS and ultimately affecting yeast viability and fermentation performance. Also, it is important to highlight the synergistic stress effect promoted by the simultaneous presence of lignocellulose-derived inhibitors and IS. Dealing with new engineering strategies such as evolutionary engineering, and further evaluating the general stress response mechanisms, ROS tolerance, and cell cycle arrest at molecular level is of utmost importance to fully comprehend and overcome the effect caused by IS on yeast cells. Furthermore, triggering different stress response mechanisms on yeast cells, especially those related with antioxidant activities (e.g. glutathione, thioredoxin and/or methionine-based scavenging systems) or those preventing programed cell death (e.g. *BIR1* and/or *STF2*) seems to be crucial to obtain novel and more robust yeasts strains for the lignocellulosic industry.

## Supplementary information

Supplementary Information

## Data Availability

All data generated or analyzed during this study and the links to the corresponding databases are included in this published article (and its Supplementary Information files).
