# Supplementary material for: Insoluble solids at high concentrations repress yeast’s response against stress and increase intracellular ROS levels
Source: Sci Rep. 2019 Aug 22;9:12236. doi: 10.1038/s41598-019-48733-w (PMC6706384; doi:10.1038/s41598-019-48733-w)
Supplement: Supplementary file 1 — Supplementary Information [file 41598_2019_48733_MOESM1_ESM.pdf]

Scientific Reports

Insoluble solids at high concentrations repress yeast's response against stress and increase intracellular ROS levels

Antonio D. Moreno[1*], Cristina González-Fernández[2], Mercedes Ballesteros[1,2], Elia Tomás-Pejó[2]

[1] *CIEMAT, Department of Energy, Biofuels Unit, 28040 Madrid, Spain*

[2] *IMDEA Energy Institute, Biotechnological Processes for Energy Production Unit, 28935 Móstoles, Spain.*

*Correspondence to: Antonio D. Moreno, CIEMAT, Department of Energy, Biofuels Unit, Avda. Complutense 40, 28040 Madrid, Spain. Phone: +34 91 346 6054. E-mail: david.moreno@ciemat.es

Supplementary Table S1

Table S1. Upregulated and downregulated genes with molecular function unknown

Gene (Systematic name)	Brief description	Log2-fold change
**Upregulated genes:**		
**ECM1**	Pre-ribosomal factor involved in 60S ribosomal protein subunit export	**0.9**
**SEO1**	Putative permease	**1.5**
**YBL028C**	Protein of unknown function that may interact with ribosomes	**0.7**
**PAU9**	Protein of unknown function	**1.9**
**MIC10**	Conserved component of the MICOS complex	**0.8**
**YCR051W**	Putative protein of unknown function; non-essential gene	**1.0**
**YDL012C**	Tail-anchored plasma membrane protein with a conserved CYSTM module; possibly involved in response to stress	**0.7**
**KNH1**	Protein with similarity to Kre9p; required for propionic acid resistance	**1.1**
**RAD59**	Protein involved DNA double-strand break repair; repairs breaks in DNA during vegetative growth via recombination and single-strand annealing	**1.1**
**YDL121C**	Putative protein of unknown function; non-essential gene	**0.8**
**YDR034C-A**	Putative protein of unknown function	**1.9**
**BCP1**	Essential protein involved in nuclear export of Mss4p, which plays a role in actin cytoskeleton organization and vesicular transport	**1.2**
**IRC22**	Protein of unknown function; non-essential gene	**0.7**
**GTT3**	Protein of unknown function may be involved in glutathione metabolism	**0.6**
**SCC4**	Subunit of cohesin loading factor (Scc2p-Scc4p); involved in establishing sister chromatid cohesion during double-strand break repair via phosphorylated histone H2AX	**1.1**
**YER156C**	Putative protein of unknown function; expression is copper responsive and downregulated in strains deleted for MAC1, a copper-responsive transcription factor	**0.9**
**FMP10**	Putative protein of unknown function	**0.8**
**YER186C**	Putative protein of unknown function	**1.0**
**YFR035C**	Putative protein of unknown function	**1.1**
**YGL101W**	Protein of unknown function; non-essential gene	**0.9**
**MTO1**	Mitochondrial protein	**1.0**

**VHT1**	High-affinity plasma membrane H+-biotin (vitamin H) symporter	**0.8**
**YGR125W**	Putative protein of unknown function	**0.6**
**BNS1**	Protein of unknown function	**1.2**
**YGR266W**	Protein of unknown function; protein abundance increases in response to DNA replication stress	**1.2**
**CIA2**	Component of cytosolic iron-sulfur protein assembly (CIA) machinery; forms the CIA targeting complex with Cia1p and Met18p that directs Fe-S cluster incorporation into a subset of proteins involved in methionine biosynthesis, DNA replication and repair, transcription, and telomere maintenance	**1.1**
**AXL2**	Integral plasma membrane protein; required for axial budding in haploid cells	**0.8**
**AIM20**	Protein of unknown function; overexpression causes cell cycle delay or arrest	**0.9**
**YJL047C-A**	Putative protein of unknown function	**1.1**
**ALB1**	Shuttling pre-60S factor; involved in the biogenesis of ribosomal large subunit	**0.8**
**CYC1**	Cytochrome c, isoform 1; electron carrier of mitochondrial intermembrane space that transfers electrons from ubiquinone-cytochrome c oxidoreductase to cytochrome c oxidase during cellular respiration	**2.7**
**YKL068W-A**	Putative protein of unknown function	**1.8**
**DPH2**	Protein required for synthesis of diphthamide	**1.1**
**SRP40**	Nucleolar serine-rich protein; role in preribosome assembly or transport; may function as a chaperone of small nucleolar ribonucleoprotein particles (snoRNPs)	**1.0**
**PTR2**	Integral membrane peptide transporter; mediates transport of di- and tri-peptides	**1.6**
**MIM2**	Mitochondrial protein required for outer membrane protein import	**1.1**
**YML018C**	Protein of unknown function; non-essential gene	**0.9**
**SUR7**	Plasma membrane protein, component of eisosomes	**1.5**
**MIX17**	Mitochondrial intermembrane space protein; required for normal oxygen consumption; protein abundance increases in response to DNA replication stress	**0.8**
**AIM34**	Protein of unknown function	**1.5**
**FMP42**	Putative protein of unknown function	**1.0**
**YNL095C**	Putative protein of unknown function; non-essential gene	**1.6**
**CWC25**	Splicing factor required for the first step of pre-mRNA splicing; heat-stable protein	**0.8**
**YOL014W**	Putative protein of unknown function	**1.3**
**YOL019W**	Protein of unknown function	**0.8**

**ZEO1**	Peripheral membrane protein of the plasma membrane; regulates the cell integrity pathway mediated by Pkc1p and Slt2p	**0.8**
**CYT1**	Cytochrome c1; component of the mitochondrial respiratory chain	**1.4**
**YOR072W-B**	Putative protein of unknown function	**1.7**
**YTM1**	Constituent of 66S pre-ribosomal particles; required for maturation of the large ribosomal subunit	**1.0**
**SFG1**	Nuclear protein putative transcription factor; required for growth of superficial pseudohyphae but not for invasive pseudohyphal growth	**1.1**
**CIR2**	Putative ortholog of human ETF-dH; may have a role in oxidative stress response	**1.9**
**TYW1**	Iron-sulfer protein required for synthesis of Wybutosine modified tRNA; induction by Yap5p in response to iron provides protection from high iron toxicity; overexpression results in increased cellular iron	**0.8**
**NIP7**	Nucleolar protein required for 60S ribosome subunit biogenesis	**1.0**
**Downregulated genes:**		
**MAK16**	Essential nuclear protein; maintenance of killer	**-1.0**
**FRT2**	Tail-anchored ER membrane protein of unknown function; functionally related to TCP1	**-2.0**
**FUN19**	Non-essential protein of unknown function; expression induced in response to heat stress	**-1.1**
**YAR023C**	Putative integral membrane protein	**-1.1**
**YAR029W**	Member of DUP240 gene family but contains no transmembrane domains (protein localized to the cytoplasm)	**-1.0**
**MOH1**	Protein of unknown function, essential for stationary phase survival	**-1.6**
**YBL111C**	Helicase-like protein encoded within the telomeric Y' element; relocalizes from mitochondrion to cytoplasm upon DNA replication stress	**-1.3**
**YRO2**	Protein with a putative role in response to acid stress (protein localized to the mitochondrion and detected in plasma membrane in the presence of acetic acid)	**-2.6**
**YBR085C-A**	Protein of unknown function	**-1.0**
**VID24**	GID Complex regulatory subunit; vacuolar import and degradation	**-0.8**
**AIM3**	Protein that inhibits barbed-end actin filament elongation; altered inheritance rate of mitochondria	**-1.8**
**YBR138C**	Cytoplasmic protein of unknown function	**-2.2**
**YSW1**	Protein required for normal prospore membrane formation	**-1.3**
**SDS24**	Protein involved in cell separation during budding	**-2.2**
**MTC4**	Cytoplasmic protein of unknown function	**-1.7**
**YBR285W**	Putative protein of unknown function	**-1.2**

**FUS1**	Membrane protein localized to the shmoo tip that regulates the termination of mating projection growth; has a role in cortical protein anchoring	**-1.0**
**GID7**	Subunit of GID Complex that binds directly to central component Vid30p	**-0.9**
**HSP30**	Negative regulator of the H(+)-ATPase Pma1p involved in the response to various stresses such as heat, osmotic stress, and DNA damage; also involved in negative regulation of ATPase activity	**-2.0**
**GPM2**	Homolog of Gpm1p phosphoglycerate mutase; may be non-functional	**-2.3**
**USO1**	Essential protein involved in vesicle-mediated ER to Golgi transport; binds membranes and functions during vesicle docking to the Golgi; required for assembly of the ER-to-Golgi SNARE complex	**-2.5**
**PEX19**	Chaperone and import receptor for newly-synthesized class I PMPs; binds peroxisomal membrane proteins (PMPs) in the cytoplasm and delivers them to the peroxisome for subsequent insertion into the peroxisomal membrane	**-1.2**
**BUG1**	Cis-golgi localized protein involved in ER to Golgi transport; forms a complex with the mammalian GRASP65 homolog, Grh1p	**-1.2**
**MSS2**	Peripherally bound inner membrane protein of the mitochondrial matrix; involved in membrane insertion of C-terminus of Cox2p	**-1.9**
**YDL109C**	Putative lipase; involved in lipid metabolism; non-essential gene	**-1.4**
**IWR1**	RNA polymerase II transport factor, conserved from yeast to humans; also has a role in transporting RNA polymerase III into the nucleus	**-1.1**
**SAS10**	Subunit of U3-containing Small Subunit (SSU) processome complex; involved in production of 18S rRNA and assembly of small ribosomal subunit; disrupts silencing when overproduced	**-1.5**
**MSH5**	Protein of the MutS family; forms a dimer with Msh4p that facilitates crossovers between homologs during meiosis	**-1.1**
**RTN2**	Reticulon protein; involved in nuclear pore assembly and maintenance of tubular ER morphology; promotes membrane curvature; regulates the ER asymmetry-induced inheritance block during ER stress; role in ER-derived peroxisomal biogenesis	**-1.5**
**FMP45**	Integral membrane protein localized to mitochondria; required for sporulation and maintaining sphingolipid content	**-1.6**
**HBT1**	Shmoo tip protein, substrate of Hub1p ubiquitin-like protein	**-1.2**
**ADY3**	Protein required for spore wall formation; mediates assembly of the LEP complex, formation of the ring-like structure via interaction with spindle pole body components and prospore membrane maturation	**-1.6**
**GAL3**	Transcriptional regulator; involved in activation of the GAL genes in response to galactose	**-1.1**
**RAD61**	Subunit of a complex that inhibits sister chromatid cohesion; also negatively regulates chromosome condensation	**-1.5**
**PST1**	Cell wall protein that contains a putative GPI-attachment site; secreted by regenerating protoplasts; upregulated by cell wall damage via disruption of FKS1	**-1.1**
**YOS9**	ER quality-control lectin; participates in efficient ER retention of misfolded proteins by recognizing them and delivering them to Hrd1p	**-1.2**

**RRG1**	Protein of unknown function; required for vacuolar acidification and mitochondrial genome maintenance	**-1.3**
**DOS2**	Protein of unknown function	**-1.7**
**SLU7**	RNA splicing factor; required for ATP-independent portion of 2nd catalytic step of spliceosomal RNA splicing	**-2.0**
**DOP1**	Golgi-localized, leucine-zipper domain containing protein; involved in endosome to Golgi transport, organization of the ER, establishing cell polarity, and morphogenesis	**-0.6**
**VPS64**	Protein required for cytoplasm to vacuole targeting of proteins	**-1.6**
**RAV2**	Subunit of RAVE complex (Rav1p, Rav2p, Skp1p)	**-0.9**
**DON1**	Meiosis-specific component of the spindle pole body	**-1.0**
**MTH1**	Negative regulator of the glucose-sensing signal transduction pathway	**-1.5**
**RAD34**	Protein involved in nucleotide excision repair (NER)	**-1.8**
**ESC2**	Sumo-like domain protein; prevents accumulation of toxic intermediates during replication-associated recombinational repair; roles in silencing, lifespan, chromatid cohesion and the intra-S-phase DNA damage checkpoint	**-1.9**
**YDR391C**	Putative protein of unknown function; possibly involved in zinc homeostasis; Bdf1p-dependent transcription induced by salt stress	**-1.1**
**HEH2**	Inner nuclear membrane (INM) protein	**-1.1**
**PRP3**	Splicing factor; component of the U4/U6-U5 snRNP complex	**-2.2**
**EMI2**	Non-essential protein of unknown function implicated in sporulation and transcription regulation; required for transcriptional induction of the early meiotic-specific transcription factor IME1; required for sporulation	**-1.1**
**KRE28**	Subunit of a kinetochore-microtubule binding complex; required for sister chromatid bi-orientation and kinetochore binding of SAC components	**-2.4**
**FIT1**	Mannoprotein that is incorporated into the cell wall	**-2.0**
**IRC4**	Protein of unknown function	**-1.3**
**RGI1**	Protein of unknown function; involved in energy metabolism under respiratory conditions; protein abundance is increased upon intracellular iron depletion or in response to DNA replication stress	**-1.7**
**SPI1**	GPI-anchored cell wall protein involved in weak acid resistance	**-1.1**
**YER158C**	Protein of unknown function; potentially phosphorylated by Cdc28p	**-0.8**
**SLO1**	Protein interacting with Arl3p	**-1.2**
**IES1**	Subunit of the INO80 chromatin remodeling complex; relocalizes to the cytosol in response to hypoxia	**-0.8**
**LAM5**	Putative sterol transfer protein with a role in intracellular sterol transport, by similarity with Lam6p	**-0.9**

**COS4**	Endosomal protein involved in turnover of plasma membrane proteins; predicted to have a role in the multivesicular body sorting pathway	**-0.9**
**YFL065C**	Putative protein of unknown function; induced by treatment with 8-methoxypsoralen and UVA irradiation	**-1.4**
**FAR7**	Protein involved in recovery from pheromone-induced cell cycle arrest	**-1.4**
**YFR016C**	Protein of unknown function	**-1.5**
**ERJ5**	Type I membrane protein with a J domain; required to preserve the folding capacity of the endoplasmic reticulum; loss of the non-essential ERJ5 gene leads to a constitutively induced unfolded protein response	**-0.9**
**RIM8**	Protein involved in proteolytic activation of Rim101p; part of response to alkaline pH	**-1.0**
**YGL081W**	Putative protein of unknown function; non-essential gene; interacts genetically with CHS5, a gene involved in chitin biosynthesis	**-1.1**
**RRT6**	Putative protein of unknown function; non-essential gene identified in a screen for mutants with increased levels of rDNA transcription	**-1.1**
**NUT1**	Component of the RNA polymerase II mediator complex; mediator is required for transcriptional activation and also has a role in basal transcription	**-1.0**
**VID30**	Central component of GID Complex, involved in FBPase degradation; required for association of Vid vesicles and actin patches in vacuole import and degradation pathway; shifts the balance of nitrogen metabolism toward glutamate production	**-1.2**
**YGL262W**	Putative protein of unknown function	**-1.1**
**STF2**	Protein involved in resistance to desiccation stress; exhibits antioxidant properties, and its overexpression prevents ROS accumulation and apoptosis	**-1.1**
**SEC9**	t-SNARE protein required for secretory vesicle-plasma membrane fusion	**-1.7**
**MTL1**	Putative plasma membrane sensor; involved in cell integrity signaling and stress response during glucose starvation and oxidative stress	**-0.9**
**MCO32**	Putative protein of unknown function	**-1.6**
**YGR066C**	Putative protein of unknown function	**-1.8**
**YGR130C**	Component of the eisosome with unknown function	**-1.6**
**ECL1**	Protein of unknown function; overexpression increases oxygen consumption and respiratory activity while deletion results in reduced oxygen consumption under conditions of caloric restriction	**-0.9**
**YHR022C**	Putative protein of unknown function; non-essential gene	**-1.4**
**NMD2**	Protein involved in the nonsense-mediated mRNA decay (NMD) pathway	**-1.6**
**RTC3**	Protein of unknown function involved in RNA metabolism	**-1.2**
**YHR140W**	Putative integral membrane protein of unknown function	**-1.2**
**LIN1**	Non-essential component of U5 snRNP	**-2.6**

**KEL1**	Protein required for proper cell fusion and cell morphology	**-1.3**
**PEX18**	Peroxin; peroxisomal protein involved in the import of proteins into the peroxisomal matrix	**-1.3**
**VID28**	GID Complex subunit, serves as adaptor for regulatory subunit Vid24p; vacuolar import and degradation	**-0.9**
**YIL055C**	Putative protein of unknown function	**-1.8**
**YRB2**	Protein of unknown function; involved in nuclear processes of the Ran-GTPase cycle; involved in nuclear protein export; contains Ran Binding Domain and FxFG repeats	**-1.2**
**PCI8**	Possible shared subunit of Cop9 signalosome (CSN) and eIF3	**-1.5**
**SPO22**	Meiosis-specific protein essential for chromosome synapsis; involved in synaptonemal complex assembly and protein sumoylation	**-1.1**
**YIL108W**	Putative metalloendopeptidase; forms cytoplasmic foci upon DNA replication stress	**-0.8**
**OM45**	Mitochondrial outer membrane protein of unknown function	**-1.3**
**FLO11**	GPI-anchored cell surface glycoprotein (flocculin); Protein involved in flocculation, coflocculation, cell adhesion during biofilm formation, and in pseudohyphal and invasive growth	**-1.5**
**INA22**	F1F0 ATP synthase peripheral stalk assembly factor; mitochondrial inner membrane protein and subunit of the INA complex (INAC) with a role in assembly of mitochondrial F1F0 ATP synthase	**-1.2**
**MGA2**	ER membrane protein involved in regulation of OLE1 transcription; inactive ER form dimerizes and one subunit is then activated by ubiquitin/proteasome-dependent processing followed by nuclear targeting	**-0.9**
**TPH3**	Putative protein of unknown function	**-1.1**
**PET130**	Protein required for respiratory growth	**-1.4**
**CHM7**	Yeast homolog of human CHMP7, localizes to the endoplasmic reticulum	**-1.8**
**NCA3**	Protein involved in mitochondrion organization	**-1.3**
**MTC1**	Protein of unknown function that may interact with ribosomes	**-0.7**
**YJL132W**	Putative protein of unknown function	**-1.0**
**YJL144W**	Cytoplasmic hydrophilin essential in desiccation-rehydration process; expression induced by osmotic stress, starvation and during stationary phase	**-1.2**
**FMP33**	Putative protein of unknown function	**-1.2**
**JJJ2**	Protein of unknown function	**-1.6**
**RCY1**	F-box protein involved in recycling endocytosed proteins; involved in recycling plasma membrane proteins internalized by endocytosis	**-1.0**
**LSO1**	Protein with a potential role in response to iron deprivation	**-1.0**

**MHO1**	Protein of unknown function	**-1.1**
**MNN14**	Putative protein of unknown function; non-essential gene	**-0.9**
**BIR1**	Subunit of chromosomal passenger complex (CPC); required for chromosome bi-orientation and for spindle assembly checkpoint activation upon reduced sister kinetochore tension; relative distribution to shortened microtubules increases upon DNA replication stress	**-1.5**
**SGM1**	Protein of unknown function; required for wild-type growth rate on galactose and mannose	**-2.3**
**YJR149W**	Putative protein of unknown function	**-1.5**
**YJR154W**	Putative protein of unknown function	**-1.1**
**DID4**	Class E Vps protein of the ESCRT-III complex; required for sorting of integral membrane proteins into lumenal vesicles of multivesicular bodies, and for delivery of newly synthesized vacuolar enzymes to the vacuole, involved in endocytosis	**-0.6**
**VPS24**	One of four subunits of the ESCRT-III complex; forms an endosomal sorting complex required for transport III (ESCRT-III) subcomplex with Did4p; involved in the sorting of transmembrane proteins into the multivesicular body (MVB) pathway	**-0.9**
**BLI1**	Subunit of the BLOC-1 complex involved in endosomal maturation	**-1.1**
**STB6**	Protein that binds Sin3p in a two-hybrid assay	**-1.4**
**RRP14**	Essential protein, constituent of 66S pre-ribosomal particles; interacts with proteins involved in ribosomal biogenesis and cell polarity	**-2.3**
**YJU2**	Essential protein required for pre-mRNA splicing	**-1.3**
**YKL107W**	Putative short-chain dehydrogenase/reductase	**-1.2**
**SBA1**	Co-chaperone that binds and regulates Hsp90 family chaperones; involved in protein folding, positive regulation of telomere maintenance via telomerase, negative regulation of DNA binding, and regulation of telomerase activity	**-0.4**
**RCI50**	Putative protein of unknown function; not required for growth of cells lacking the mitochondrial genome; SWAT-GFP and mCherry fusion proteins localize to the mitochondria	**-1.1**
**COY1**	Golgi membrane protein with similarity to mammalian CASP; genetic interactions with GOS1 (encoding a Golgi snare protein) suggest a role in Golgi function	**-1.4**
**YKR011C**	Protein of unknown function; protein abundance increases in response to DNA replication stress	**-1.0**
**YKR015C**	Putative protein of unknown function	**-1.1**
**MIC60**	Component of the MICOS complex; involved in mitochondrial cristae formation and the import of proteins into the mitochondrial intermembrane space	**-0.7**
**YKR023W**	Putative protein of unknown function; is detected in highly purified mitochondria in high-throughput studies	**-1.6**
**YKR041W**	Protein of unknown function; localizes to the mitotic spindle; its overexpression affects endocytic protein trafficking	**-1.3**

**THI73**	Putative plasma membrane permease; proposed to be involved in carboxylic acid uptake and repressed by thiamine	**-1.9**
**SNF7**	One of four subunits of the ESCRT-III complex; involved in ubiquitin-dependent protein catabolism via the multivesicular body sorting pathway, late endosome to vacuole transport, intralumenal vesicle formation, ATP export, and the cellular response to anoxia	**-1.0**
**YLR046C**	Putative membrane protein	**-0.8**
**OSW2**	Protein of unknown function reputedly involved in spore wall assembly	**-1.0**
**EMP46**	Integral membrane component of ER-derived COPII-coated vesicles	**-2.5**
**GIS3**	Protein of unknown function	**-1.3**
**AVL9**	Conserved protein involved in exocytic transport from the Golgi	**-1.5**
**YLR149C**	Protein of unknown function; non-essential gene; null mutation results in a decrease in plasma membrane electron transport; protein abundance increases in response to DNA replication stress	**-1.1**
**YLR179C**	Protein of unknown function with similarity to Tfs1p	**-0.7**
**YLR225C**	Putative protein of unknown function; non-essential gene	**-1.0**
**CMG1**	Putative protein of unknown function	**-2.0**
**IMH1**	Protein involved in vesicular transport	**-1.5**
**ATG23**	Peripheral membrane protein required for autophagy and CVT; involved in upregulation of macroautophagy, protein localization to pre-autophagosomal structure, late nucleophagy, cytoplasm-to-vacuole targeting (CVT) pathway	**-1.5**
**YML003W**	Putative protein of unknown function	**-1.2**
**ITT1**	Protein that modulates the efficiency of translation termination	**-1.1**
**COG8**	Component of the conserved oligomeric Golgi complex; involved in vesicle-mediated transport within the Golgi and CVT pathway	**-1.0**
**MSC1**	Protein of unknown function	**-1.3**
**YML131W**	Protein of unknown function	**-1.4**
**BUD22**	Protein required for rRNA maturation and ribosomal subunit biogenesis	**-1.2**
**TAP42**	Essential protein involved in the TOR signaling pathway	**-1.4**
**EIS1**	Component of the eisosome required for proper eisosome assembly; protein increases in abundance and relocalizes from plasma membrane to cytoplasm upon DNA replication stress	**-1.4**
**KAR5**	Protein required for nuclear membrane fusion during karyogamy	**-1.0**
**SOV1**	Mitochondrial protein of unknown function	**-1.1**
**YMR111C**	Protein of unknown function	**-1.0**

**SHH3**	Putative mitochondrial inner membrane protein of unknown function	**-1.0**
**YMR122W-A**	Protein of unknown function	**-0.5**
**YMR158C-A**	Putative protein of unknown function	**-2.0**
**YMR181C**	Protein of unknown function; non-essential gene	**-0.8**
**MIN3**	Putative protein of unknown function	**-1.3**
**VTI1**	Protein involved in cis-Golgi membrane traffic; SNAP receptor subunit of SNARE complex that mediates membrane fusion; integral component of Golgi membrane involved in vesicle-mediated transport and fusion, Golgi to vacuole transport, non-autophagic vacuole fusion, and macroautophagy	**-0.7**
**YMR253C**	Putative protein of unknown function; non-essential gene	**-1.2**
**VAC7**	Integral vacuolar membrane protein	**-1.2**
**YNL115C**	Putative protein of unknown function	**-0.9**
**FYV6**	Protein of unknown function; required for survival upon exposure to K1 killer toxin; proposed to regulate double-strand break repair via non-homologous end-joining	**-0.9**
**EAF7**	Subunit of the NuA4 histone acetyltransferase complex	**-1.5**
**YNL144C**	Putative protein of unknown function	**-1.3**
**YGP1**	Cell wall-related secretory glycoprotein; may be involved in adaptation prior to stationary phase entry	**-2.1**
**KAR1**	Protein involved in karyogamy during mating and spindle pole body duplication during mitosis	**-1.9**
**SQS1**	Protein that stimulates the ATPase and helicase activities of Prp43p	**-1.2**
**PDR16**	Phosphatidylinositol transfer protein (PITP); controls levels of various lipids, may regulate lipid synthesis	**-0.7**
**YTP1**	Probable type-III integral membrane protein of unknown function	**-1.0**
**KRI1**	Essential nucleolar protein required for 40S ribosome biogenesis	**-1.3**
**EGO4**	Protein of unknown function	**-3.1**
**YNR068C**	Putative protein of unknown function	**-1.3**
**TLG2**	Syntaxin-like t-SNARE; required along with VPS45 for an early step of the constitutive CVT pathway; interactions with Vps45 prevents Tlg2p degradation, and facilitates t-SNARE complex formation	**-1.6**
**DDR2**	Multi-stress response protein; expression is activated by a variety of xenobiotic agents and environmental or physiological stresses	**-1.0**
**IZH4**	Membrane protein involved in zinc ion homeostasis; possible role in sterol metabolism; protein increases in abundance and relocalizes from nucleus to ER upon DNA replication stress	**-1.0**

**PEP12**	Target membrane receptor (t-SNARE); for vesicular intermediates traveling between the Golgi apparatus and the vacuole; controls entry of biosynthetic, endocytic, and retrograde traffic into the prevacuolar compartment; syntaxin	**-0.9**
**TMC1**	AN1-type zinc finger protein, effector of proteotoxic stress response	**-0.6**
**SLD7**	Protein with a role in chromosomal DNA replication	**-1.5**
**RTS2**	Basic zinc-finger protein	**-1.5**
**YOR097C**	Putative protein of unknown function; non-essential gene	**-1.1**
**SIA1**	Protein of unassigned function; involved in activation of the Pma1p plasma membrane H+-ATPase by glucose	**-1.1**
**SLK19**	Kinetochore-associated protein; equired for chromosome segregation and kinetochore clustering; required for normal segregation of chromosomes in meiosis and mitosis	**-2.2**
**RRP36**	Component of 90S preribosomes	**-1.1**
**SNU66**	Component of the U4/U6.U5 snRNP complex	**-1.0**
**FIT2**	Mannoprotein that is incorporated into the cell wall; involved in the retention of siderophore-iron in the cell wall	**-0.6**
**FIT3**	Mannoprotein that is incorporated into the cell wall; involved in the retention of siderophore-iron in the cell wall	**-1.0**
**YOR389W**	Putative protein of unknown function; expression regulated by copper levels	**-1.5**
**ATG29**	Autophagy-specific protein; involved in macroautophagy, late nucleophagy, mitochondrion degradation and piecemeal microautophagy of nucleus; required for recruiting other ATG proteins to the pre-autophagosomal structure (PAS)	**-1.3**
**UIP4**	Protein that interacts with Ulp1p	**-1.8**
**YPL191C**	Protein of unknown function	**-1.2**
**APL5**	Delta adaptin-like subunit of the clathrin associated protein complex	**-1.1**
**YPL247C**	Putative protein of unknown function	**-1.1**
**YPL277C**	Putative protein of unknown function; gene expression regulated by copper levels	**-1.5**
**YPL278C**	Putative protein of unknown function; gene expression regulated by copper levels	**-1.1**
**THP3**	Protein that may have a role in transcription elongation; possibly involved in splicing based on pre-mRNA accumulation defect for many intron-containing genes	**-1.1**
**YPR127W**	Putative pyridoxine 4-dehydrogenase	**-0.9**

Data obtained from Saccharomyces Genome Database (https://www.yeastgenome.org)

Supplementary Figure S1

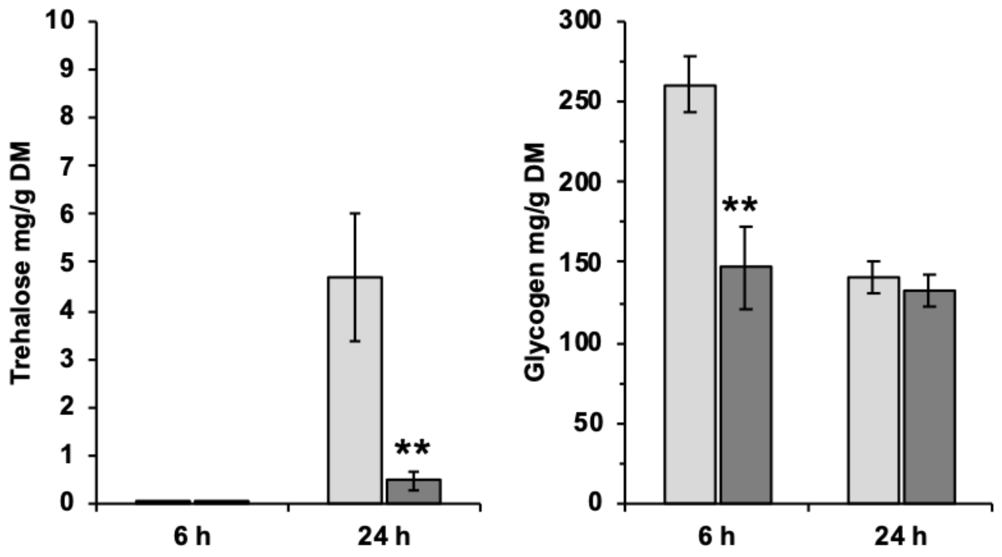

Figure S1. Intracelular trehalose and glycogen concentration after 6 and 24 h of fermentation in the presence of 0% (light grey) and 40% (dark grey) IS (w/w). Significant differences: ** $P < 0.01$
